# Using Optical Coherence Tomography and Intravascular Ultrasound Imaging to Quantify Coronary Plaque Cap Stress/Strain and Progression: A Follow-Up Study Using 3D Thin-Layer Models

**DOI:** 10.3389/fbioe.2021.713525

**Published:** 2021-08-23

**Authors:** Rui Lv, Akiko Maehara, Mitsuaki Matsumura, Liang Wang, Caining Zhang, Mengde Huang, Xiaoya Guo, Habib Samady, Don. P. Giddens, Jie Zheng, Gary S. Mintz, Dalin Tang

**Affiliations:** ^1^ School of Biological Science and Medical Engineering, Southeast University, Nanjing, China; ^2^ The Cardiovascular Research Foundation, Columbia University, New York, NY, United States; ^3^ School of Science, Nanjing University of Posts and Telecommunications, Nanjing, China; ^4^ Department of Medicine, Emory University School of Medicine, Atlanta, GA, United States; ^5^ The Wallace H. Coulter Department of Biomedical Engineering, Georgia Institute of Technology, Atlanta, GA, United States; ^6^ Mallinckrodt Institute of Radiology, Washington University, St. Louis, MO, United States; ^7^ Mathematical Sciences Department, Worcester Polytechnic Institute, Worcester, MA, United States

**Keywords:** coronary plaque, plaque progression prediction, patient-specific coronary plaque models, vulnerable plaque, optical coherence tomography-based coronary models

## Abstract

Accurate plaque cap thickness quantification and cap stress/strain calculations are of fundamental importance for vulnerable plaque research. To overcome uncertainties due to intravascular ultrasound (IVUS) resolution limitation, IVUS and optical coherence tomography (OCT) coronary plaque image data were combined together to obtain accurate and reliable cap thickness data, stress/strain calculations, and reliable plaque progression predictions. IVUS, OCT, and angiography baseline and follow-up data were collected from nine patients (mean age: 69; m: 5) at Cardiovascular Research Foundation with informed consent obtained. IVUS and OCT slices were coregistered and merged to form IVUS + OCT (IO) slices. A total of 114 matched slices (IVUS and OCT, baseline and follow-up) were obtained, and 3D thin-layer models were constructed to obtain stress and strain values. A generalized linear mixed model (GLMM) and least squares support vector machine (LSSVM) method were used to predict cap thickness change using nine morphological and mechanical risk factors. Prediction accuracies by all combinations (511) of those predictors with both IVUS and IO data were compared to identify optimal predictor(s) with their best accuracies. For the nine patients, the average of minimum cap thickness from IVUS was 0.17 mm, which was 26.08% lower than that from IO data (average = 0.23 mm). Patient variations of the individual errors ranged from ‒58.11 to 20.37%. For maximum cap stress between IO and IVUS, patient variations of the individual errors ranged from ‒30.40 to 46.17%. Patient variations of the individual errors of maximum cap strain values ranged from ‒19.90 to 17.65%. For the GLMM method, the optimal combination predictor using IO data had AUC (area under the ROC curve) = 0.926 and highest accuracy = 90.8%, vs. AUC = 0.783 and accuracy = 74.6% using IVUS data. For the LSSVM method, the best combination predictor using IO data had AUC = 0.838 and accuracy = 75.7%, vs. AUC = 0.780 and accuracy = 69.6% using IVUS data. This preliminary study demonstrated improved plaque cap progression prediction accuracy using accurate cap thickness data from IO slices and the differences in cap thickness, stress/strain values, and prediction results between IVUS and IO data. Large-scale studies are needed to verify our findings.

## Introduction

Cardiovascular disease is a serious threat to human life and health. Atherosclerotic plaques often rupture without warning, leading to acute cardiovascular syndrome. It is commonly agreed that plaques prone to rupture (also called thin-cap fibroatheroma, TCFA) normally have three main characteristics: large lipid-rich necrotic core, higher prevalence of macrophage infiltration in the fibrous cap, and a fibrous cap with thickness less than 65 μm (Kolodgie et al., 2001). Among the three, cap thickness may be one of the more closely monitored and critical factors for assessing plaque stress, strain, and vulnerability. Quantification of fibrous cap thickness and cap stress/strain conditions plays an important role in plaque progression prediction and vulnerability assessment, which in turn has clinical importance in monitoring disease development and patient management.

Considerable efforts have been made in investigating plaque progression using image-based patient follow-up data (Yang et al., 2010; Samady et al., 2011; Stone et al., 2011; Stone et al., 2012; Hung et al., 2016; Wang et al., 2018). Plaque vessel wall thickness change was often used as a measure for plaque progression. In most available patient follow-up data (follow-up time around 1 year), plaque vessel wall thickness changes were mostly under 100 µm in a year (Yang et al., 2010). With intravascular ultrasound (IVUS) imaging resolution at 150–200 µm and magnetic resonance imaging (MRI) resolution at 200–300 μm, it is extremely challenging to quantify and predict plaque progression with acceptable reliability and accuracy. With its superior resolution at about 10 μm, optical coherence tomography (OCT) is getting acceptance in research community and clinical practices. OCT is able to detect the thin fibrous cap of vulnerable plaques (Kume et al., 2006). However, due to its limited penetration, OCT is not able to “see” the whole vessel. Several groups have combined IVUS and OCT in detecting and analyzing vulnerable plaques, and impressive results have been reported (Sawada et al., 2008; Fujii et al., 2015; Zanchin et al., 2020). As an initial effort, a modelling approach combining IVUS and OCT was introduced by Guo et al. (2019) for cap thickness quantification, more accurate cap stress/strain calculations, and plaque progression prediction. With IVUS and OCT combined, the merged IVUS + OCT (IO) data include the whole vessel from IVUS and accurate cap thickness from OCT. The accurate and reliable image data provide a solid base for further vulnerable plaque investigations. In particular, we should be able to trust cap thickness change measured by OCT and use that to obtain some reliable progression prediction results.

The goal of this study is to extend the initial effort by Guo et al. (2019) to a multipatient study. IVUS, OCT, and angiography data from nine patients with follow-up were used to generate IVUS and IVUS + OCT (IO) slices, where IO slices were made by combining IVUS and OCT segmented contours together. Since cap thickness is one of the most critical and most closely monitored measurable plaque features linked to plaque vulnerability, it was selected as the target for our prediction study. A total of 114 matched (baseline and follow-up) slices were obtained to quantify their cap thickness differences between IVUS and OCT data and their changes between baseline and follow-up. 3D thin-layer models were constructed using both IVUS and IO slices to obtain cap stress and strain data. Two prediction methods (a generalized linear mixed model (GLMM) and least squares support vector machine (LSSVM) method) were used to predict cap thickness changes using nine morphological and mechanical risk factors. Prediction accuracies by all combinations (total 511) of those predictors with both IVUS and IO data were compared to identify the optimal predictor(s) with their best accuracies.

## Materials and Methods

### Data Acquisition and Processing

IVUS, OCT, and angiography data including three epicardial coronary vessels were collected at Cardiovascular Research Foundation (CRF) from patients with coronary heart diseases between April 2017 and November 2018 using the protocol approved by the local institute, and informed consent forms were obtained from the patients. Nine lipid-rich plaques were identified from nine patients with good-quality baseline and follow-up data matched for our analysis (average follow-up days: 251). These patients had stable angina pectoris and underwent percutaneous coronary intervention (PCI). Patients with acute coronary syndrome, severe calcified lesion, chronic total occlusion, or chronic kidney disease (Cr > 1.5 mg/ dl) were excluded. Patient demographic data are shown in Table 1. IVUS examination was performed after 0.2 mg of intracoronary nitroglycerin. The IVUS catheter was advanced as far as possible using a commercially available IVUS system: a 40 MHz IVUS catheter (OptiCross, Boston Scientific Corporation, Natick, Massachusetts) with motorized pullback at 0.5 mm/ s. OCT images were acquired with ILUMIEN OPTIS System and Dragonfly or Dragonfly JP Imaging Catheter (St. Jude Medical, Westford, Massachusetts).

**TABLE 1:** Patient demographic and clinical information. F: female; M: male; BL: baseline; FU: follow-up; HT: hypertension; DM: diabetes mellitus; HL: hyperlipidemia.

Patient ID	Age	Sex	Vessel segment	Segment length (cm)	BP (mmHg)	Slice number	Diagnosis history	Follow-up days
P1	80	F	RCA	3.9	71–138	60	HT DM	304
P2	70	M	RCA	2.6	84–155	42	HT	273
P3	65	F	RCA	19.5	63–149	41	DM	220
P4	66	M	LCX	2.0	89–150	49	DM	290
P5	73	M	LCX	2.2	55–150	45	HT HL	248
P6	74	F	LAD	2.3	62–151	58	HT DM HL	244
P7	62	F	LAD	1.9	79–117	48	HL	195
P8	61	M	LCX	16.4	78–128	40	HT DM HL	283
P9	72	M	LCX	2.9	80–143	42	HT DM HL	272

**FIGURE 1:**
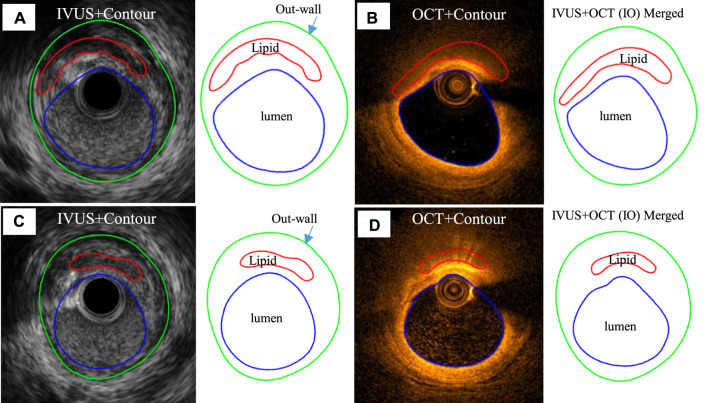
Sample IVUS and merged IVUS + OCT (IO) slices showing segmentation and merging at baseline and follow-up. **(A)** IVUS slice and its segmented contours at baseline. **(B)** Corresponding OCT slice and IVUS + OCT (IO) slice at baseline. **(C)** IVUS slice and its segmented contour plot at follow-up. **(D)** Corresponding OCT slice and IVUS + OCT (IO) contour plot at follow-up. Outwall contours of IO slices are taken from IVUS.

Coregistration of IVUS and OCT slices was performed by using fiduciary points such as side branches, bifurcations, and calcifications with the assistance of quantitative coronary angiography (QCA) images. IVUS and OCT frames at baseline and follow-up were also matched to quantify changes of plaque morphology and cap thickness using the same matching techniques. Three plaque compositions were considered in segmentation of IVUS and OCT images: lipid-rich necrotic core (short form, lipid) and calcification and other vessel tissue (fibrotic, fibro-fatty, etc.). Segmentation was performed by ImageJ 1.52 v software. For IVUS images, we used manual segmentation. For OCT images, we used the combination of manual segmentation and algorithms. Details of the segmentation methods were previously published (Lv et al., 2020). Small-size plaque components were neglected for simplicity. Matched IVUS and OCT slices were combined together to form IVUS + OCT (IO) slices with OCT providing accurate cap thickness quantifications, while IVUS providing vessel outboundary contours not visible from OCT (Lv et al., 2020). Sample IO and IVUS slices with segmented contours are given in Figure 1 showing the merging process.

**FIGURE 2:**
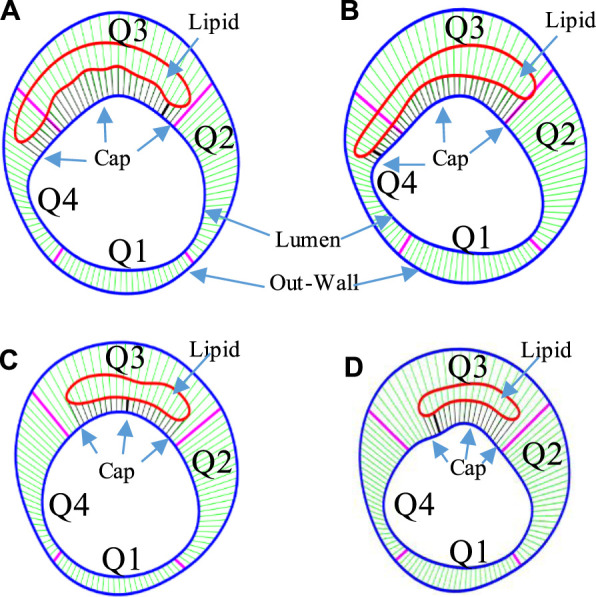
The Four-Quarter Even-Spacing method with which cap thickness was defined and calculated. Cap thickness differences between IVUS and IO slices and between baseline and follow-up are shown. **(A)** IVUS segmented contours with lumen–outwall connection lines at baseline. **(B)** Corresponding IVUS + OCT (IO) segmented contours with lumen–outwall connection lines at baseline. **(C)** IVUS slice with connection lines at follow-up. **(D)** Corresponding IVUS + OCT (IO) slice with connection lines at follow-up. Minimum cap thickness was marked using bold black.

IVUS and corresponding IO slices at baseline and follow-up were matched, and 114 matched lipid-slices were selected for analysis. Each slice (IVUS and IO) was divided into 100 parts using 100 evenly spaced lumen points and a Four-Quarter Even-Spacing method so that vessel and cap thickness could be calculated properly and recorded for analysis (Wang et al., 2019). Figure 2 illustrates our Four-Quarter Even-Spacing method with which cap thickness was defined and calculated. It also shows clear cap thickness difference between IVUS and IO slices and between baseline and follow-up. Nine morphological and mechanical risk factors were selected as our predictors to predict cap thickness change from baseline to follow-up: lumen area (LA), plaque area (PA) which is defined as the area between the vessel outwall (see Figure 2) and the lumen contour, plaque burden (PB) defined as the ratio of the plaque area over the area inside the vessel outwall, minimum cap thickness (MinCapT), mean cap thickness (MeanCapT), maximum cap stress (MaxCapS), mean cap stress (MeanCapS), maximum cap strain (MaxCapSn), and mean cap strain (MeanCapSn). Their values were calculated from baseline IVUS and IO slices. Values of cap-related predictors were collected from related cap lumen points (see Figure 2). For better clarity, the distance between the lumen point and its corresponding wall point was defined as the wall thickness. The connecting line from the lumen contour to the edge of the lipid near the lumen was defined as cap thickness (CapT). For a given slice, MinCapT was defined as the minimum of all the fibrous cap point thicknesses for the slice. When analysis was performed at patient level, MinCapT was defined as the minimum of all fibrous cap point thicknesses in all lipid slices of the patient. Slice and patient MeanCapT were defined the same way. Maximum and mean values of stress and strain predictors were calculated similarly. Data for all predictors were collected, calculated, and saved for prediction model use. The work of morphology calculation was accomplished by MATLAB (MATLAB R2018a, MathWorks, United States).

### The 3D Thin-Layer Biomechanical Model

**FIGURE 3:**
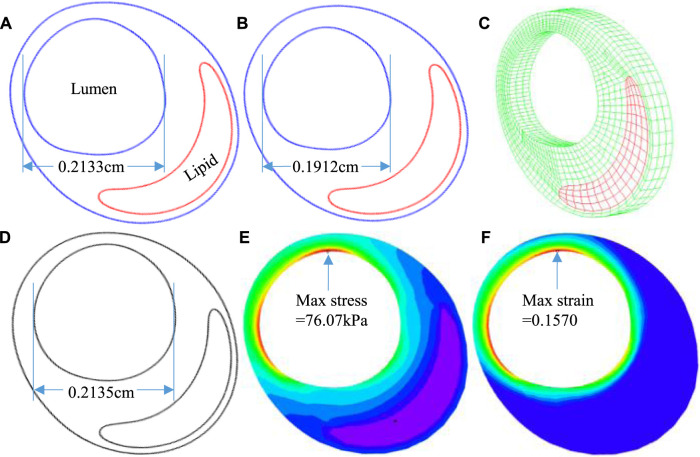
The 3D thin-layer model construction process. **(A)** An *in vivo* IVUS + OCT (IO) slice. **(B)** The slice after shrinking. **(C)** The 3D thin-layer model with a thickness added. **(D)** The slice with load-added matching *in vivo* slice morphology. **(E)** Plaque wall stress under minimum pressure. **(F)** Plaque wall strain under minimum pressure.

For the 114 matched IVUS and IO slices containing a lipid-rich core with fibrous cap, a thin-slice thickness (0.5 mm) was used to make the 3D thin-layer models (see Figure 3). Since IVUS and OCT data were acquired under *in vivo* condition when the vessel was under stretched and loaded conditions, a 5% axial shrink–stretch combined with our circumferential preshrink process was performed to obtain *in vivo* slice morphology with proper plaque stress/strain when 5% axial stretch and pressure condition were imposed (Wang et al., 2019). Details of the preshrink procedure are described in our previous studies (Yang et al., 2010; Wang et al., 2015). Vessel tissue was assumed to be hyperelastic, anisotropic, nearly incompressible, and homogeneous (Guo et al., 2018). Plaque components (lipid core and calcification) were assumed to be hyperelasic, isotropic, and nearly incompressible. A modified Mooney–Rivlin material model was used to describe the material properties of vessel tissue with the strain-energy density function given as follows (Wang et al., 2018):

$$W_{iso}=c_{1}\left(I_{1}-3\right)+c_{2}\left(I_{2}-3\right)+D_{1}\left[\exp\left(D_{2}\left(I_{1}-3\right)\right)-1\right],$$ (1)

$$W_{aniso}=W_{iso}+\frac{K_{1}}{K_{2}}\left\{\exp\left[K_{2}{\left(I_{4}-1\right)}^{2}\right]-1\right\},$$ (2)

where $I_{1}=\sum \left(C_{ii}\right)$ and $I_{2}=\frac{1}{2}\left(I_{1}^{2}-C_{ij}C_{ij}\right)$, in which $\;I_{1}$ and $I_{2}$ are the first and second invariants of right Cauchy–Green deformation tensor $C=\left(C_{ij}\right)=F^{T}F=\left(F_{ii}\right)=\left(\partial x_{i}/\partial a_{j}\right)$, $\left(x_{i}\right)$ is the current position, $\left(a_{j}\right)$is the original position, and $I_{4}=C_{ij}{\left(n_{c}\right)}_{i}{\left(n_{c}\right)}_{j}$, where $n_{c}$ is the unit vector in the circumferential direction of the vessel and $c_{1}$, $\;c_{2}$, $D_{1}$, $D_{2}$, $K_{1}$, and $K_{2}$ are the material parameters. Material constants of the isotropic Mooney–Rivlin model from the existing literature were used. Lipid: $c_{1}$ = 0.5 kPa, $c_{2}$ = 0 kPa, $D_{1}$ = 0.5 kPa, and $D_{2}$ = 1.5; calcification: $c_{1}$ = 92 kPa,$\;c_{2}$ = 0 kPa, $D_{1}$ = 36 kPa, and $D_{2}$ = 2.0; vessel/fibrous tissue: $c_{1}$ = ‒262.76 kPa, $c_{2}$ = 22.9 kPa,$\;D_{1}$ = 125.9 kPa, $D_{2}$ = 2.0, $K_{1}$ = 7.19 kPa, and $K_{2}$ = 23.5 (Guo et al., 2018; Guo et al., 2019).

**FIGURE 4:**
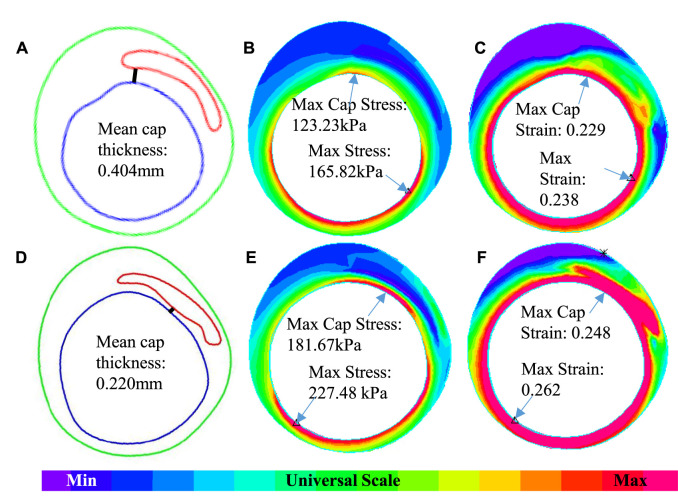
An example showing the differences of cap thickness, maximum cap stress, and maximum cap strain in the cap region and maximum stress and strain on the whole lumen for IVUS + OCT (IO) and IVUS. **(A**–**C)** IO slice—contour, stress, and strain plots. **(D**–**F)** IVUS slice—contour, stress, and strain plots.

The 3D thin-layer models were solved by finite element software ADINA 9.0 (Adina R & D, Watertown, MA, United States) following our established procedures (Wang et al., 2019). Nonlinear incremental iterative procedures were used to solve the models. Mesh analysis was performed by refining mesh density by 10% until changes of solutions became less than 2%. Figure 4 provides plots of a sample slice contour with cap thickness and stress and strain distributions to show the cap thickness and stress/strain differences between IVUS and IO models. Cap thickness, plaque stress, plaque strain, plaque cap stress, and cap strain were extracted and saved for analysis.

### Prediction Models and Strategy

Nine morphological and mechanical risk factors were selected as our predictors to predict cap thickness change from baseline to follow-up (see Data Acquisition and Processing for descriptions): LA, PA, PB, MinCapT, MeanCapT, maximum cap stress (MaxCapS), mean cap stress (MeanCapS), maximum cap strain (MaxCapSn), and mean cap strain (MeanCapSn). As the prediction target variable, Δ(MinCapT) is defined as

Δ(MinCapT)=ΔMinCapT=MinCapT at T2−MinCapT at T1, (3)

where T1 = baseline and T2 = follow-up. Δ(MinCapT) of IO was used as the benchmark for model prediction verifications. For a given slice, its binary labels for ΔMinCapT were defined as

$$Slice\;Label\left(\Delta MinCapT\right)=\{\begin{array}{c} 1,if\;\Delta MinCapT<0,\\ -1,if\;\Delta MinCapT\geq 0, \end{array}$$ (4)

where Slice Label = 1 means the lipid plaque is progressive (getting worse), while Slice Label = ‒1 means the lipid plaque is nonprogressive.

A 5-fold cross-validation strategy with generalized linear mixed model (GLMM) and least squares support vector machine (LSSVM) methods were adopted to calculate prediction accuracy for each single predictor and all combinations of the nine predictors and quantify prediction accuracy differences using IO and IVUS slice values (Wang et al., 2015). The GLMM model is defined as

$${\text{y}}_{ij}=E\left({\text{y}}_{ij}|b_{j}\right)+{\text{ε}}_{ij},$$ (5)

$$\log it\left(E\left(y_{ij}|b_{j}\right)\right)=\beta_{0}+\beta_{1}x1_{ij}+\beta_{2}x2_{ij}+\ldots +b_{j},$$ (6)

where ${\text{y}}_{ij}$ is the binary response of ΔMinCapT and $E\left({\text{y}}_{ij}\text{|}b_{j}\right)=P\left({\text{y}}_{ij}=1|b_{j}\right)$ is the probability on the i^th^ slice of the j^th^ patient. logit(x)=log[x/(1−x)] is the binomial link function. $x1_{ij}$, $x2_{ij}$, etc. are the risk factors, which were used to predict plaque cap progression. ${\text{β}}_{0}$, ${\text{β}}_{1}$, etc. are the fixed-effect coefficients, and $b_{j}$ and ${\text{ε}}_{ij}$ are the random-effect terms and the random error terms of GLMM. R function glmmPQL was used to estimate the term values by fitting GLMM.

The LSSVM method used Gaussian radial basis function as kernel function, fitted all the samples with least square error, and used the steepest descent method to find the optimal parameters. Radial basis function is given by

$$K\left(x,z\right)=\exp\left(-\frac{x-z^{2}}{\sigma^{2}}\right),$$ (7)

where $\sigma^{2}$ is the square-coordinated kernel width. MATLAB (MATLAB R2018a, MathWorks, United States) was used to adjust the parameters and complete the prediction work.

All 114 IO and IVUS slices were randomly divided into 5 subgroups, 4 as training subgroups and 1 as test subgroup. The training subgroups were used for model fitting. The test subgroup was used to test the model, and dichotomous prediction is performed. Each subgroup was used once, and the testing results from the five subgroups were combined together as the final result to give prediction accuracy. The binary responses for the measures of cap thickness changes (ΔMinCapT) were adopted to find the best predictor(s). If fibrous cap thickness decreases, it was positive (labeled 1); meanwhile, increase was negative (labeled ‒1). The classified result is judged by accuracy (Acc), sensitivity (Sen), specificity (Spe), and area under the ROC curve (AUC). Accuracy is the probability of correctly predicted slices being detected in all test groups. Sensitivity is the probability of detecting a plaque progression slice in the plaque progression test subgroup. Specificity is the probability of the correct slice being detected in the nonplaque progression test subgroup. The AUC was used to assess the predictive power of each predictor.

### Statistical Analysis

MinCapT, MeanCapT, MaxCapS, MeanCapS, MaxCapSn, and MeanCapSn from IO and IVUS models were compared to get differences. Errors by IVUS data and models were calculated using IO results as baseline (gold standard). The Kolmogorov–Smirnov tests were performed to check data normality. Analysis of variance, paired t-tests, and Mann–Whitney U test were used to check if the differences were statistically significant. A *p* value < 0.05 was considered statistically significant.

## Results

### IVUS Cap Thickness Data Had Large Errors Compared to OCT Data

Table 2 lists minimum and mean cap thickness values from nine patients based on 114 slices at baseline. Mean cap thickness (MeanCapT) was the average of point-to-point cap thickness for every patient. The average of MinCapT from IVUS was 0.17 mm, which was 26.09% lower than that from IO data (average = 0.23 mm). Patient variations of the individual errors ranged from ‒58.11 to 20.37%, with mean ± SD = ‒20.70 ± 30.32%. Negative error value means IVUS cap thickness values were lower than those from IO values. The average of MeanCapT from IVUS was 0.38 mm, which was 2.56% lower than that from IO data. Patient variations of the individual errors ranged from ‒30.32 to 25.50%, with mean ± SD = 0.16 ± 20.86%. Patient five had the biggest error (in absolute value) for both MinCapT (‒58.11%) and MeanCapT (‒30.32%). Patient six had maximum MinCapT (0.324 mm) based on IO slice, but its IVUS slice had MinCapT 0.141 mm with error ‒56.48%.

**TABLE 2:** Summary of minimum and mean cap thickness from 9 patients based on 114 slices at baseline. Unit for fibrous cap thickness: mm.

Patient	MinCapT			MeanCapT		
	IO	IVUS	Error (%)	IO	IVUS	Error (%)
Patient 1	0.162	0.195	20.37	0.251	0.315	25.50
Patient 2	0.242	0.190	−21.49	0.385	0.379	−1.56
Patient 3	0.271	0.284	4.80	0.390	0.447	14.62
Patient 4	0.317	0.135	−57.41	0.536	0.451	−5.86
Patient 5	0.222	0.093	−58.11	0.376	0.262	−30.32
Patient 6	0.324	0.141	−56.48	0.602	0.499	−17.11
Patient 7	0.181	0.144	−20.44	0.325	0.382	17.54
Patient 8	0.127	0.124	−2.36	0.273	0.230	−15.75
Patient 9	0.228	0.239	4.82	0.348	0.433	24.43
Max	0.324	0.284	20.37	0.602	0.499	25.50
Min	0.127	0.093	−58.11	0.251	0.230	−30.32
Mean ± SD	0.23 ± 0.07	0.17 ± 0.06	-20.70 ± 30.32	0.39 ± 0.11	0.38 ± 0.09	0.16 ± 20.86

### Cap Stress and Strain From IVUS and IO Data Have Large Differences and the Differences Have Large Patient Variations

Table 3 summarizes mean and maximum cap stress values from the nine patients based on simulation results from 114 3D thin-layer models. The average of IO MeanCapS of the 114 slices was 67.15 kPa. The average MeanCapS from IVUS slices was 73.44 kPa, which was 9.37% higher than that from IO models. The modest error value could be misleading since positive and negative individual errors canceled against each other in the average. Patient variations of the individual errors for MeanCapS ranged from ‒37.68 to 44.25%, with mean ± SD = 13.93 ± 29.69%. The average of MaxCapS of the 114 IO slices was 84.36 kPa, compared to 88.23 kPa from IVUS slices, which was 4.59% higher than that from IO models. Patient variations of the individual errors ranged from ‒30.40 to 46.17%, with mean ± SD = 7.24 ± 26.47%. Patientwise comparisons of IO and IVUS cap stress indicates that Patient four had the highest error for MeanCapS and MaxCapS (44.25 and 46.17%, respectively).

**TABLE 3:** Summary of mean and maximum cap stress values for the plaques of 9 patients. Unit for stress: kPa.

Patient	MeanCapS			MaxCapS		
	IO	IVUS	Error (%)	IO	IVUS	Error (%)
Patient 1	71.77	44.73	−37.68	78.65	54.74	−30.40
Patient 2	70.76	71.53	1.09	82.14	81.29	−1.03
Patient 3	90.49	91.83	1.48	101.82	100.95	−0.85
Patient 4	48.77	70.35	44.25	73.47	107.39	46.17
Patient 5	71.89	95.88	33.37	95.45	113.03	18.42
Patient 6	69.64	82.94	19.10	97.99	106.47	8.65
Patient 7	85.58	68.24	−20.26	112.25	78.89	−29.72
Patient 8	60.91	86.09	41.34	68.01	94.19	38.49
Patient 9	34.58	49.34	42.68	49.43	57.08	15.48
Max	90.49	95.88	44.25	112.25	113.03	46.17
Min	34.58	44.73	−37.68	49.43	54.74	−30.40
Mean ± SD	67.15 ± 17.27	73.44 ± 17.81	13.93 ± 29.69	84.36 ± 19.48	88.23 ± 21.62	7.24 ± 26.47

Table 4 summarizes mean and maximum cap strain values from the nine patients from our 114 3D thin-layer models. The average of IO MeanCapSn of the 114 slices was 0.15 which is the same as that from IVUS slices. However, patient variations of the individual errors ranged from ‒22.92 to 29.63%, with mean ± SD = 4.33 ± 16.10%. The average of MaxCapSn of the 114 IO slices was 0.17, which again was the same as that from the IVUS slices. Patient variations of the individual errors ranged from ‒19.90 to 17.65%, with mean ± SD = 0.11 ± 13.94%. Patientwise comparisons of IO and IVUS mean cap strain indicated that Patient nine had the highest error in 29.63% under MeanCapSn and Patient seven had the lowest error in ‒19.90% under MaxCapSn. Figure 4 gives an example to show the differences of mean cap thickness, maximum cap stress, and maximum cap strain in the cap region and maximum stress and strain on the whole lumen for IO and IVUS.

**TABLE 4:** Summary of mean and maximum cap strain values for 9 patients.

Patient	MeanCapSn			MaxCapSn		
	IO	IVUS	Error (%)	IO	IVUS	Error (%)
Patient 1	0.144	0.111	−22.92	0.154	0.125	−18.83
Patient 2	0.189	0.176	−6.88	0.215	0.192	−10.70
Patient 3	0.175	0.177	1.14	0.191	0.190	−0.52
Patient 4	0.149	0.172	15.44	0.202	0.217	7.43
Patient 5	0.065	0.073	12.31	0.084	0.094	11.90
Patient 6	0.177	0.185	4.52	0.211	0.212	0.47
Patient 7	0.169	0.151	−10.65	0.201	0.161	−19.90
Patient 8	0.159	0.185	16.35	0.171	0.194	13.45
Patient 9	0.108	0.140	29.63	0.136	0.160	17.65
Max	0.189	0.185	29.63	0.215	0.217	17.65
Min	0.065	0.073	−22.92	0.084	0.094	−19.90
Mean ± SD	0.15 ± 0.04	0.15 ± 0.04	4.33 ± 16.10	0.17 ± 0.04	0.17 ± 0.04	0.11 ± 13.94

### Cap Thickness Progression Prediction Using GLMM: Comparison Between IO and IVUS Results

**FIGURE 5:**
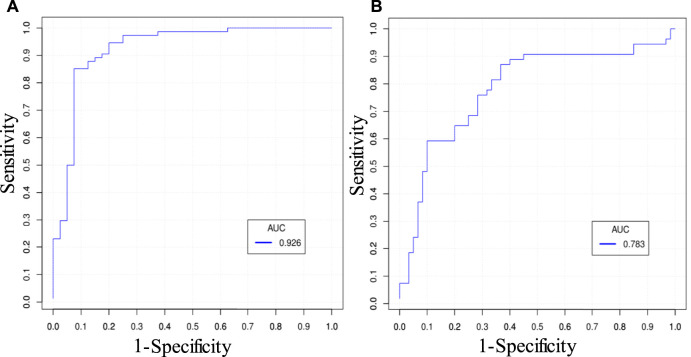
ROC curves of the best combinations of predictors predicting ΔMinCapT. **(A)** ROC curve using IO data. The best combination is given by PA + MinCapT + MeanCapS + PB. **(B)** ROC curve using IVUS data. The best combination is given by PA + MinCapT + MeanCapT + MeanCapS + MeanCapSn.

There were altogether 511 (2^9–1) combinations using nine predictors. Table 5 gives prediction results by two best combinations and nine single predictors for ΔMinCapT using IO and IVUS data based on the GLMM method, respectively. Values for prediction accuracy (Acc), specificity (Spe), and area under the ROC curve (AUC) are given. The optimal combination predictor among all 511 predictors using IO data was the combination of MinCapT, MeanCapS, PB, and PA with the highest AUC = 0.926 and highest accuracy = 90.8% for IO data, which compared favourably with AUC = 0.783 and Acc = 74.6% using IVUS data. The best single predictor was PA with the highest AUC value in IO prediction results with AUC = 0.868 and accuracy = 85%, compared to AUC = 0.637 and accuracy = 64.0% using IVUS data. The best prediction accuracy of IO using combinations was 16.2% higher than that of IVUS (90.8 *vs*. 74.6%). The AUCs for the best combinations of predictors using IVUS and IO data are given in Figure 5. For the predictor using IO data, the best combination is given by PA + MinCapT + MeanCapS + PB. For IVUS data, the best combination is given by PA + MinCapT + MeanCapT + MeanCapS + MeanCapSn.

**TABLE 5:** ΔMinCapT prediction results using the GLMM method. AUC: area under the ROC curve; Acc: accuracy; Sen: sensitivity; Spe: specificity. Bold indicates the best value.

IO prediction results					IVUS prediction results					
Predictor	Acc	Sen	Spe	AUC	Predictor	Acc	Sen	Spe	AUC	
PA + MinCapT + MeanCapS + PB	**0.908**	0.851	0.925	**0.926**	PA + MinCapT + MeanCapT + MeanCapS + MeanCapSn	**0.746**	0.870	0.633	**0.783**	
PA + MinCapT + MeanCapS	0.892	0.865	0.900	0.926	MaxCapS + MeanCapS + MaxCapSn + MeanCapSn	0.737	0.870	0.617	0.778	
PA	0.850	0.851	0.850	**0.868**	PA	0.640	0.463	0.800	0.637	
MinCapT	0.753	0.870	0.717	0.854	MinCapT	0.746	0.926	0.583	0.704	
MaxCapSn	**0.863**	0.743	0.900	0.847	MaxCapSn	0.632	0.870	0.417	0.627	
MeanCapT	0.782	0.778	0.783	0.843	MeanCapT	**0.746**	0.944	0.567	**0.774**	
PB	0.653	0.986	0.550	0.822	PB	0.693	0.833	0.567	0.711	
MaxCapS	0.653	0.986	0.550	0.814	MaxCapS	0.693	0.926	0.483	0.708	
MeanCapSn	0.653	0.986	0.550	0.788	MeanCapSn	0.649	0.870	0.450	0.635	
MeanCapS	0.653	0.986	0.550	0.786	MeanCapS	0.728	0.815	0.650	0.716	
LA	0.743	0.611	0.783	0.699	LA	0.667	0.593	0.733	0.645	

### Cap Thickness Progression Prediction Using LSSVM: Comparison Between IO and IVUS Results

Using the LSSVM method to predict ΔMinCapT, Table 6 gives prediction results by two best combinations and nine single predictors using IO and IVUS data, respectively. The optimal combination predictor (best AUC value) among all 511 predictors using IO data was the combination of PA, PB, MinCapT, and MeanCapS with AUC = 0.838 and accuracy = 75.7%. The best predictor for IVUS data was a combination of MeanCapT, MaxCapS, and MeanCapSn with AUC = 0.780 and accuracy = 69.6%. The best single predictor was MinCapT in IO prediction results with AUC = 0.804 and accuracy = 76%, compared to AUC = 0.725 and accuracy = 71.5% using IVUS data. The best prediction accuracy of IO using combinations was 6.1% higher than that of IVUS (75.7 vs. 69.6%).

**TABLE 6:** ΔMinCapT prediction results using the LSSVM method. AUC: area under the ROC curve; Acc: accuracy; Sen: sensitivity; Spe: specificity. Bold indicates the best value.

IO prediction results					IVUS prediction results				
Predictor	Acc	Sen	Spe	AUC	Predictor	Acc	Sen	Spe	AUC
PA + MinCapT + MeanCapS + PB	**0.757**	0.793	0.725	**0.838**	MeanCapT + MaxCapS + MeanCapSn	**0.696**	0.749	0.649	**0.780**
PA + MinCapT + MeanCapS + MeanCapT	0.717	0.716	0.718	0.837	MeanCapT + MaxCapS + MeanCapS + PB	0.706	0.752	0.664	0.771
MinCapT	**0.760**	0.737	0.792	**0.804**	MinCapT	**0.715**	0.748	0.670	**0.725**
MeanCapT	0.741	0.670	0.825	0.764	MeanCapT	0.613	0.639	0.578	0.613
MeanCapSn	0.622	0.652	0.586	0.633	MeanCapSn	0.594	0.663	0.499	0.615
PA	0.605	0.642	0.555	0.608	PA	0.570	0.702	0.388	0.518
LA	0.545	0.619	0.456	0.552	LA	0.565	0.684	0.401	0.544
MeanCapS	0.541	0.476	0.601	0.544	MeanCapS	0.618	0.737	0.454	0.608
MaxCapS	0.590	0.436	0.728	0.543	MaxCapS	0.644	0.711	0.551	0.626
PB	0.576	0.726	0.371	0.537	PB	0.608	0.660	0.535	0.600
MaxCapSn	0.548	0.635	0.445	0.506	MaxCapSn	0.633	0.685	0.563	0.652

### Combining Morphological and Mechanical Factors Improved Prediction Accuracies

**FIGURE 6:**
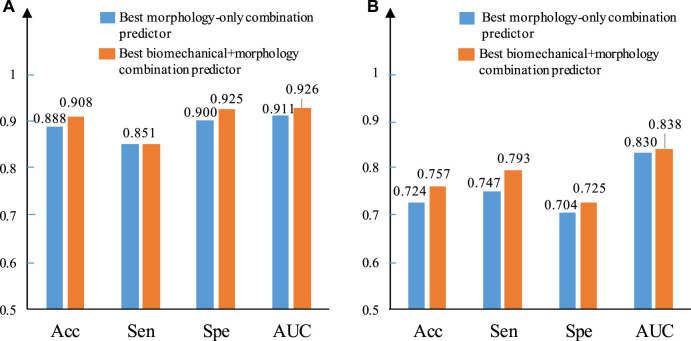
Comparison between the best morphology–only combined predictor and the best biomechanical + morphology combined predictor using IO data based on ΔMinCapT prediction results. **(A)** IO prediction result based on GLMM. **(B)** IO prediction result based on LSSVM. AUC: area under the ROC curve; Acc: accuracy; Sen: sensitivity; Spe: specificity.

Figure 6 provides bar plots comparing prediction results using only morphological predictors and using combinations of morphological and mechanical predictors. Results from GLMM and LSSVM methods using IO data are shown. For the GLMM method, combining biomechanical and morphological factors helped to improve prediction accuracy from 88.8 to 90.8%. The AUC value improved from 0.911 to 0.926. Sensitivity did not show improvement. Specificity improved from 0.9 to 0.925. The LSSVM method showed similar improvements, while overall performance was not as good as that from the GLMM method.

## Discussion

### Significance of Accurate Cap Thickness Data for Plaque Progression Assessment

In order to predict plaque progression, one simple truth is that we must be able to acquire the progression data with enough accuracy to predict. With IVUS imaging resolution at 150–200 μm and MRI resolution at 200–300 μm, and with the fact that plaque vessel wall thickness changes were mostly under 100 μm in a year, well below imaging resolutions (Yang et al., 2010), it is extremely challenging to quantify and predict plaque progression with acceptable reliability and accuracy. OCT comes with a superior 10–15 μm resolution, and it gives us hope that we may get accurate data to work with. However, OCT is limited by its penetration and cannot get the whole vessel cross section. Combining IVUS and OCT provides one approach where we have the whole cross section from IVUS and accurate cap thickness from OCT. Since the whole cross section is primarily from IVUS with its resolution limitation, it remains to be questionable to use vessel wall thickness as the measure for plaque progression. In this study, cap thickness was selected as the measure for plaque progression. While it does not give complete information for plaque changes, it does come with the accuracy from OCT and it is one of the most critical plaque features affecting plaque stress/strain conditions. The well-known cap thickness threshold value (65 μm) of vulnerable plaques is usually used as an important criterion for morphological classification (Virmani et al., 2002, Virmani et al., 2006; Cardoso and Weinbaum, 2014; Fenning and Wilensky, 2014; Saba et al., 2014; Arbab-Zadeh and Fuster, 2015; Goncalves et al., 2015; Bala et al., 2018; Yonetsu and Jang, 2018; Zhao et al., 2018; Porcu et al., 2020). For the first time, the progression prediction models were based on image data with resolution good enough to quantify thin cap thickness and the predicted target parameter (cap thickness) has values that are accurate and reliable. With that, prediction accuracies should be more reliable and meaningful.

Cap thickness data from OCT (and IO slices) are considered and used as the gold standard. Therefore, comparison of IVUS and IO data and prediction results is to show the errors (uncertainties) by IVUS images when we have “true” cap thickness obtained by OCT. It should be noted that the average of MeanCapT from IVUS was only 2.56% lower than that from IO data, while patient variations of the individual errors ranged from ‒30.32 to 25.50%, with mean ± SD = 0.16 ± 20.86%. The average of MinCapT from IVUS was 0.17 mm, which was 26.09% lower than that from IO data (average = 0.23 mm). Patient variations of the individual MinCapT errors were even worse: it ranged from ‒58.11 to 20.37%, with mean ± SD = ‒20.70 ± 30.32%. These data indicated that cap thickness data from IVUS have high uncertainties and should be interpreted and used with caution.

### Comparison of Prediction Results by GLMM and LSSVM Methods and by IVUS and IO Data

While image data accuracy is the base of any prediction research, selection of prediction methods is also of great interest. The use of prediction models requires their stability, simplicity, extendibility, and good adaptability. The logistic regression model is one of the commonly used models for plaque prediction (Samady et al., 2011; Stone et al., 2012). GLMM is commonly used in clinical medicine, which can be seen as an extended form of GLM without the requirement to meet the normal distribution of dependent variable, and has the characteristics of GLM method at the same time including fixed effect and random effect. Because risk factors were extracted from the slices, the quantitative factors between adjacent slices were neither random nor independent of each other, and GLMM could take this into account. Artificial intelligence (AI) and various machine leaning methods have been introduced to perform predictions with limited success (Han et al., 2020; Kay et al., 2020). For this work, 114 slice data constitute a relatively small data set in general machine learning method applications. SVM is a machine learning method suitable for small samples. LSSVM is a support vector machine optimized by the least squares method. The function maps the space where the samples are mapped to a higher dimensional space, which helps to recognize the targeted group/classification (Guo et al., 2019). Both GLMM and LSSVM methods have the potential to further improve the accuracy of predictions. In this study, the quantitative index based on fibrous cap thickness is more direct and simple. Based on R language, the GLMM method has fast debugging and good stability. The prediction result of GLMM’s optimal combination predictor was better than that of LSSVM (AUC 0.926 vs*.* 0.830).

Prediction accuracy using IO data (AUC = 0.926 and accuracy = 90.8%, by GLMM) is clearly better that using IVUS data (AUC = 0.783 and accuracy = 74.6%, by GLMM). The comparison seems to be unfair since the prediction target ΔMinCapT was quantified using IO data. Our purpose is not to show that IO data are better since it was used as the gold standard in this study. The purpose of the comparison is to show the impact of IVUS data uncertainty on prediction accuracy. The differences of prediction accuracies using LSSVM were smaller, as shown in Table 6 (AUC: 0.838 vs*.* 0.780; accuracy: 83 vs*.* 69.6%).

### Cap Stress and Strain Differences Between IVUS and IO Models

Mechanical forces, especially cap stress and strain, play an important role in plaque progression and vulnerability assessment. Cap thickness differences between IVUS and IO data led to large cap stress/strain differences. It is worth noting that overall averaged cap stress and strain values had very modest differences between IO and IVUS data, while individual differences ranged from about −40% to + 40%. Patient variations of the individual errors for mean cap stress (MeanCapS) ranged from ‒37.68 to 44.25%, with mean ± SD = 13.93 ± 29.69%. For maximum cap stress which could be closely related to potential plaque rupture, patient variations of the individual errors ranged from ‒30.40 to 46.17%, with mean ± SD = 7.24 ± 26.47%. For cap strain comparisons, patient variations of individual errors of mean cap strain (MeanCapSn) ranged from ‒22.92 to 29.63%, with mean ± SD = 4.33 ± 16.10%. Patient variations of the individual errors for MinCapSn ranged from ‒19.90 to 17.65%, with mean ± SD = 0.11 ± 13.94%. Errors for strain comparisons were smaller than those from stress comparisons.

### Combining Biomechanical and Morphological Factors Could Improve Prediction Accuracy

Combination of morphology and mechanical predictors may give better prediction accuracy than using morphology only or single-factor predictors which was seen again in this work. Using the GLMM method, there is an improvement in accuracy from 88.8 to 90.8%, in Sen + Spe from 0.751 to 1.776 and in AUC value from 0.911 to 0.926 based on IO data (Figure 6A). The corresponding IVUS combined predictors shows the same trend with MaxCapSn, which helps the combination of LA and MeanCapT increase the AUC from 0.756 to 0.778. Figure 6B illustrates a similar result using the LSSVM method. Combining morphological factors and mechanical factors demonstrated a certain ability not to be ignored in plaque progression prediction.

### Further Plaque Prediction Strategies

Plaque progression is a long and complex process, and it involves many factors. Data from multichannels should be collected. And, ideally, more time points (i.e., follow-up 1, follow-up 2, etc.) would be desirable and may give better results. However, those patient studies are very expensive, especially for this IVUS + OCT studies. A one-year follow-up is a typical timeframe researchers use for plaque progression investigations (Samady et al., 2011; Stone et al., 2012; D’Ascenzo et al., 2021). OCT has an accuracy of 10–15 µm and can detect relatively small changes in cap thickness. Compared to other publications for plaque progression research, we tend to believe that better accuracy from OCT should lead to more accurate and reliable predictions.

### Limitations and Future Directions

Automation of simulation modelling has always been a goal. A 3D thin-layer model helps move forward this process. However, there is still a need for further improvement. Patient-specific vessel material properties were not available for this study, and parameters from our previous studies using VH-IVUS and Cine-IVUS were used (Guo et al., 2019). 3D thin-layer models did not take into account the effects of fluid. The modest improvement of combining morphological and mechanical factors could also be due to the fact that flow shear stress is not included in the predictor list. Biochemical indicators such as hyperlipidemia or diabetes mellitus were not considered. Further refinement of the prediction models is needed.

One major limitation of this study is its small sample size. Clinical significance of our results in predicting plaque progression and regression is limited by the small patient size. Large-scale studies are needed to further verify our findings and bring our research scheme closer to clinical practice. It should be noted that data needed for this study are extremely hard to get because the data set included IVUS and OCT at both baseline and follow-up. Not only IVUS and OCT are very expensive, but also they are invasive procedures. While it is relatively easier to get patients to participate at baseline since they need to do that for their diagnosis anyway, it is much harder to recruit patients to do those invasive imaging procedures (IVUS and OCT) mainly for research purpose. It involves additional risk with only limited benefits to patients. Most insurances do not have coverage for such procedures. So, financial support becomes a limiting factor. These are practical difficulties in the data acquisition front. We will keep trying to get more data, improve our models, and move closer to clinical implementations.

Another major limitation is that predictors in this study only included morphological and biomechanical factors from structure-only models. Atherosclerosis is a pathophysiological process. While plaque rupture may be the result of the mechanical failure of the fibrous cap, there is a cascade of mechanisms involved in the thinning of the cap or the prevention of such thinning that are critical to monitor, so critical that is what is currently monitored in the clinical setting. Additional factors such as plaque erosion, inflammation, blood conditions, genomic factors, and many others should be explored to improve our models and prediction accuracies.

With the same context about vulnerable plaque research in biomechanical setting and current clinical practices, plaque stenosis severity remains to be the commonly quantified, monitored, and used in clinical diagnosis and treatment decision-making (stenting or conservative medication). Researchers have tried in recent years seeking better risk indicators including plaque components (therefore cap thickness) and plaque stress/strain conditions using more advanced imaging modalities and mechanical models. Cap thickness does play an important role in plaque stress/strain calculations. Yet, there are still considerable gaps between research findings and actual clinical implementations.

## Data Availability

The original contributions presented in the study are included in the article/supplementary material; further inquiries can be directed to the corresponding author.
